# A non-invasive method for light-inducible knockout across all cell types in mouse subcutaneous adipose tissue

**DOI:** 10.1080/21623945.2026.2671558

**Published:** 2026-05-12

**Authors:** Erica de Sousa, Magdalena Blaszkiewicz, Kristy Townsend

**Affiliations:** Department of Neurological Surgery, The Ohio State University College of Medicine and Wexner Medical Center, Columbus, OH, USA

**Keywords:** Optogenetics, adipose tissue, light-inducible Cre, photoactivatable (PA)-Cre, tissue knockout, BDNF, obesity, development

## Abstract

Cre recombination is a widely used technique for mechanistic insights in physiology and disease. However, available constitutive and inducible Cre systems present challenges that can be prohibitive for some study designs. For example, Cre expression can result in cell types targeted across numerous tissues and organs, or when a gene is expressed across multiple cell types in a tissue, Cre-Lox restricted knockout will not enable ablation across an entire tissue or organ. Photoactivatable Cre (PA-Cre) systems enable temporally and spatially restricted gene expression control in delimited anatomical regions, typically requiring a micro-LED or fibre optic implantation. Here, we report as proof-of-concept the effective knockout of BDNF in subcutaneous adipose tissue after PA-Cre activation through external blue light illumination in awake, freely moving mice. We demonstrated that for mice with black fur, shaving can be used to anatomically limit PA-Cre activation. BDNF protein expression was decreased by 87% in the inguinal scWAT after blue light exposure, with no effect observed in the perigonadal (deep) or axillary subcutaneous (non-shaved) adipose tissues. We propose blue light induction of PA-Cre as safe and effective to study adipose tissue physiology and pathology across models. Considerations for applying this tool to future studies are also presented.

## GRAPHICAL ABSTRACT

**Figure:**
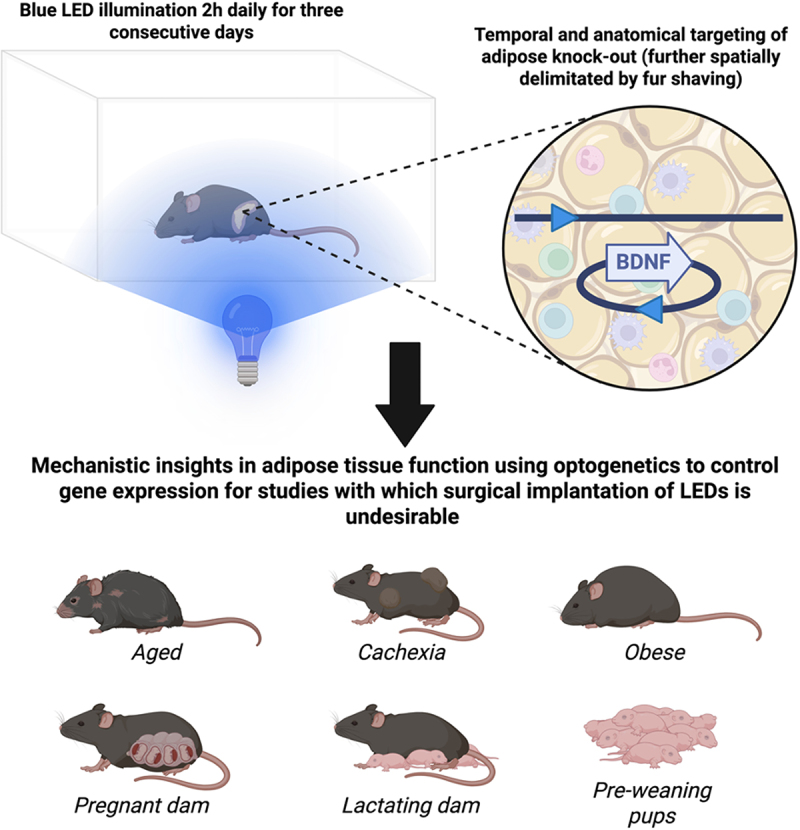

## Introduction

**Figure 1.**
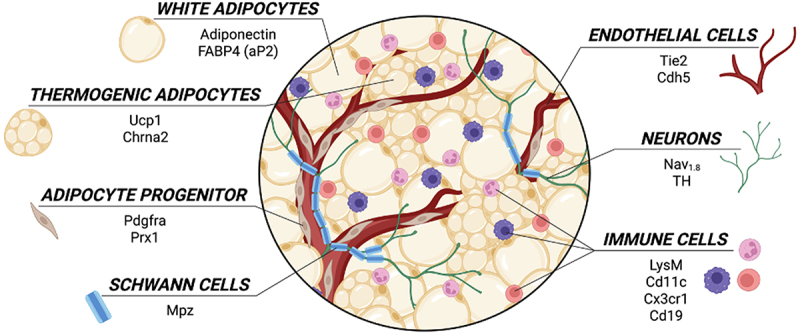
Promoters used to drive Cre expression in several cell types relevant to adipose tissue research. The white adipose tissue is composed of several cell types including adipocytes (white and thermogenic), adipocyte precursor/stem cells, endothelial cells, immune cells, neuronal axons (sensory, sympathetic) and Schwann cells. Promoters previously used to drive Cre expression in each of these cells are listed.

Critical functions in metabolic homoeostasis are carried out by adipose tissue, and mechanistic contributions of adipose molecular pathways in physiology and disease continue to be unravelled, including the tissue’s secretory activity [1–3], and roles for the tissue’s neural innervation [4–8] that provide important organ crosstalk functions. Cre recombinase has been widely applied to catalyse the recombination between loxP sites for mechanistic insights gleaned by gene knockout studies (i.e. Cre-Lox mouse models). In constitutive systems, the Cre recombinase enzyme is expressed ubiquitously (for example, ROSA26, driving gene expression in embryonic stem cells, which is useful for developmental studies [9]) or under the control of a tissue-specific promoter requiring no external inducer. Examples of Cre promoters for cells relevant for adipose tissue studies include: for mature adipocytes: adiponectin and fatty acid-binding protein 4 (Fabp4) [10]; thermogenic adipocytes: uncoupling protein 1 (Ucp1) [11,12] or cholinergic receptor nicotinic alpha 2 subunit (Chrna2) [13]; for immune cells: Cd11c [14], Cd19 [15], lysine motif (LysM) and C-X3-C motif chemokine receptor 1 (Cx3cr1) [16]; for adipocyte progenitors: platelet-derived growth factor receptor alpha (Pdgfra) [10] or paired related homeobox 1 protein (Prx1) [17]; for endothelial cells: TEK receptor tyrosine kinase (Tie2) [18] or vascular endothelial cadherin (Cdh5) [19]; for nerves: voltage-gated sodium channel 1.8 (Na_v_1.8) [20] or tyrosine hydroxylase (TH) [21]; and for Schwann cells: myelin protein zero (MPZ) [22] (summarized in Figure 1). Constitutive Cre recombinase produces a strong and reliable recombination in cell types expressing the promoter gene, but due to its constitutive expression (i.e. no temporal control), this system does not allow the study of genes whose deletion is embryonic lethal (e.g. the key microRNA processing enzyme Dicer [23]) or may cause confounding developmental effects versus adult ablation of the gene, thereby complicating the interpretation of the gene function [24].

To overcome this limitation, inducible systems were developed in which the timing of Cre expression can be controlled by a transgene that is activated by exogenously provided treatments, for example, tamoxifen for CreERT and CreERT2 systems [25], or doxycycline for tetracycline (Tet)-controlled systems [26]. In ligand-inducible systems (CreERT, CreERT2), Cre is fused with a modified oestrogen receptor ligand-binding domain. In the absence of the ligand (tamoxifen), CreERT remains in the cytosol sequestered by heat-shock proteins. After tamoxifen administration, the complex translocates to the nucleus, enabling recombination. It enables tight temporal control, but tamoxifen can alter several organs’ function through oestrogen receptor signalling [27]. Also, depending on the design of the study, tamoxifen can be toxic. For example, tamoxifen use in pregnant mice induces offspring malformations [28]. In the tetracycline-inducible system (Tet-On/Tet-Off), Cre expression is coupled to tetracycline responsive elements (TRE). These elements can be controlled by tTA (Tet-Off) or rtTA (Tet-On) trans activators, in which Cre is expressed in the absence of doxycycline or after doxycycline administration, respectively. The regulation of inducible Cre systems is dose-dependent, but since doxycycline is an antibiotic, variable confounding factors like microbiota alterations can be introduced, complicating the interpretation of data [27]. In both constitutive and inducible Cre-Lox systems, Cre expression may confound data interpretation due to expression of some genes across multiple tissues (e.g. rat-insulin promoter (Rip)-Cre used in pancreas also hits the nervous system [29,30], Ap2-Cre used to target adipocytes also targeted macrophages and neurons [31,32]). In other cases, a complete tissue knockout is desired, but numerous cell types may express the gene of interest, thus precluding the use of a Cre-based system that typically targets a single cell type around the body, or the use of viral vectors that have limited cellular tropism and rarely infect immune cells.

**Figure 2.**
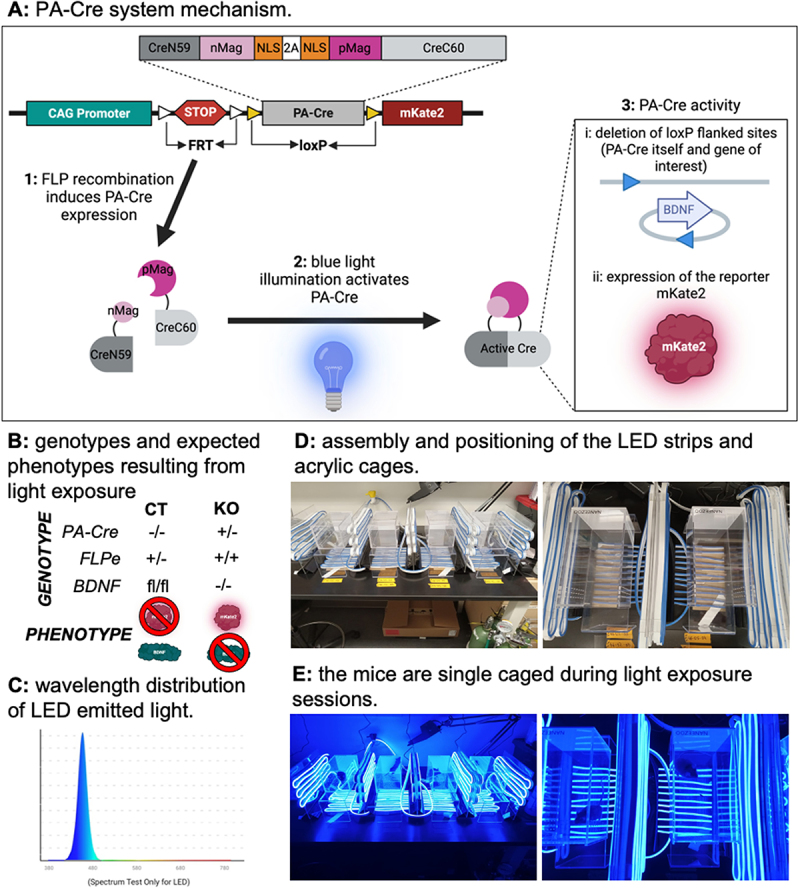
The PA-Cre system and light box structure. A: PA-Cre system elements: FLPe recombination induces PA-Cre expression, which is post-transcriptionally divided into two fragments that dimerize upon blue light exposure. After dimerization, PA-Cre deletes loxP flanked sites, removing both the target gene and its own sequence, allowing the reporter mKate2 to be expressed. B: Genotypes and phenotypes resulting from blue light exposure. BDNF is expressed across several cell types in adipose and was used as an example for this system. C: Wavelength distribution of the LED emitted light measured inside the cage. D: Assembly and positioning of the LED strips and acrylic cages. E: Mice are single caged during the light exposure sessions.

The photoactivatable Cre (PA-Cre) system enables gene editing under the control of blue light exposure, allowing precise spatiotemporal control [33]. PA-Cre is expressed after FLP-mediated excision of frt-flanked STOP cassette, then it is cleaved at the P2A self-cleaving peptide sequence, yielding two split Cre segments associated with the proteins of the Magnet system (nMag:CreN59 and pMag:CreC60). Upon light exposure, nMag and pMag dimerize, promoting Cre assembly and function. PA-Cre deletes loxP flanked regions, which include its own sequence and the gene of interest and allows the fluorescent reporter mKate2 expression (Figure 2(A)). The Magnet system was engineered from the protein Vivid, a flavin-binding photoreceptor derived from the fungus *Neurospora crassa* with absorbance spectrum peaking at 450 nm with two minor peaks at 428 and 478 nm [34]. A previous study on the visible light absorption in mouse skin found that light ranging 400–500 nm can penetrate around 2000 µm [35], corroborated by Monte Carlo simulations [36]. Skin thickness in mice varies from 211 to 671 µm [37], meaning that tissues right below the skin can be reached by blue light.

In this study, we use a floxed brain derived neurotrophic factor (BDNF) mouse as proof of concept, since it is already known that this gene is expressed across several cell types in the adipose tissue, like preadipocytes [38] and immune cells [16]. It was already shown that PA-Cre can be activated in the liver after external illumination in mice shaved on their abdominal region [33,39]. Herein, we validated this approach to target the inguinal scWAT, avoiding gene deletion in the axillary scWAT and visceral depots like perigonadal. Often this approach is desirable, for example to ablate BDNF from all cell types where it is expressed in ing-scWAT to determine impacts on depot-specific innervation and nerve functions. We also demonstrated the light induction can be limited anatomically using the animal’s fur as a shield to prevent PA-Cre activation in other regions, but this may not be as successful in mice without black fur and is an important consideration when this method is applied to future studies.

## Material and methods

### Animals

The experiments described in this article were approved by The Ohio State University IACUC under protocol 2021 A00000004-R1. Mice were housed on a 12-h light/dark cycle with chow diet and water provided ad libitum. Mouse models were generated by first crossing B6;129S-Gt(ROSA)26Sortm1(CAG-cre*,-mKate2)Yzwa/J (Rosa26^PA-Cre A20^) JAX strain #033544 to Bdnf^tm3Jae^/J JAX strain #004339 and B6.129S4-Gt(ROSA)26Sor^tm1(FLP1)Dym^/RainJ (ROSA26:FLPe knock in) JAX strain 009086 to Bdnf^tm3Jae^/J JAX strain #004339 until PA-Cre^+/-^::BDNF^fl/+^ and FLPe^+/+^::BDNF^fl/+^ genotypes were obtained. These were then bred together to produce PA-Cre^+/-^::FLPe^+/+^::BDNF^fl/fl^ animals for light-inducible knock-out of BDNF experimental groups, and PA-Cre^−/−^::FLPe^+/-^::BDNF^+/+^ littermates as controls groups. The genotypes and expected phenotypes after blue light exposure are presented in Figure 2(B).

### The light box system

The light box system optimized in our lab is comprised of easily accessible supplies. Blue LED flexible silicone strips (600 SMD2835 LEDs distributed in 10 m; 12 volts; 60 watts; 6500K colour temperature. Brand: Jo.Ko) were fixed to transparent acrylic sheets to arrange them to cover the cages in both bottom and laterals. This blue light source peaked at 460 nm with illuminance of 243 lux inside each cage (tested with AH-300 light metre, Aquahorti, Figure 2(C)). Illuminance (lux) was converted to irradiance (W/m2) using the Commission Internationale d’Eclairage (CIE) 1931 photopic luminous efficiency function (V(λ)) [40] and the peak emission wavelength of the light source. The spectral luminous efficiency value for 460 nm (V(λ) ≈ 0.06) was incorporated into the equation:

$$\mathrm{Irradiance}=\frac{\mathrm{Illuminance}}{683\times V\left(\lambda \right)}$$

Therefore, one lux is equivalent to 0.0244 W/m^2^ and the irradiance inside the light box is 5.9 W/m^2^. Each cage is an acrylic transparent reptile terrarium measuring 4 × 4 × 8 inches with ventilation holes to ensure air flux and a sliding door with a magnetic snap (brand: Naneezoo). The cages were elevated using acrylic transparent stands, ensuring their proper positioning relative to the light source, as shown in Figure 2(D).

### Photoactivatable Cre expression and induction

The mice were shaved only in the region above the inguinal adipose tissue and single caged in the light box system described above (Figure 2(E)). Each light exposure session was 2 h long, provided daily for three consecutive days. Seven days after the last blue LED illumination session, the mice were euthanized through CO2 asphyxiation followed by cervical dislocation. The axillary and inguinal subcutaneous adipose depots and the visceral perigonadal were dissected for BDNF protein expression and mKate2 reporter expression assessments.

### Western blot

Unilateral inguinal scWAT depots were quickly dissected and snap frozen in liquid nitrogen. Tissues were homogenized in RIPA buffer (ThermoFisher, cat# PI89900) supplemented with phosphatases and proteases inhibitors (Sigma Aldrich, cat# P5726, P0044 and P8340) according to the supplier’s recommendation. Protein quantification was performed using the Bradford method (Bio-Rad, cat# 5,000,006), total protein normalization was performed, and 30ug of protein per samples was loaded in a 4–20% gradient SDS gel (Bio-Rad, cat# 4,561,096) and transferred to PVDF membranes (Bio-Rad, cat# 1,620,174). To prevent non-specific antibody binding membranes were blocked for 1 h in 10% blocking reagent (Roche, cat#11921681001) in agitation at room temperature. Membranes were incubated with primary antibody anti-BDNF (Abcam, cat# ab108319) diluted 1:1000 in 5% blocking reagent overnight at 4°C on an orbital shaker. After 3 × 10 min washes in TBST and 1 × 10 min wash in TBS, the membranes were incubated with IgG HRP-conjugated secondary antibody (Cell Signalling, cat# 7074P2) diluted 1:3000 for 1 h at room temperature and detected through enhanced chemoluminescence (ThermoFisher, cat# 32,106). The blots were imaged on a Syngene G:BOX (Frederick, MD, USA).

### Immunofluorescent imaging

The other side of the three adipose depots were fixed in 2% PFA overnight at 4C for whole mount imaging according to Willows et al [41]. The tissues were incubated with the ChromoTek pan-RFP polyclonal antibody (ProteinTech, cat# pabr1) diluted 1:500 in PBS to boost mKate2 reporter signal. After washing in PBS shaking in the cold room, the tissues were incubated with secondary antibody conjugated to Alexa 647 diluted 1:500 in PBS (Invitrogen, cat# A32733). The tissues were imaged using Leica Stellaris 5 confocal microscope. Image processing was performed in Leica LASX software.

### Statistical analysis

BDNF protein expression within each tissue was compared between CT and KO mice through a t test using GraphPad Prism 10.3.1 for macOS. Data was presented as average ± SEM. A *p*-value equal or lower than 0.05 was considered significant.

## Results

**Figure 3.**
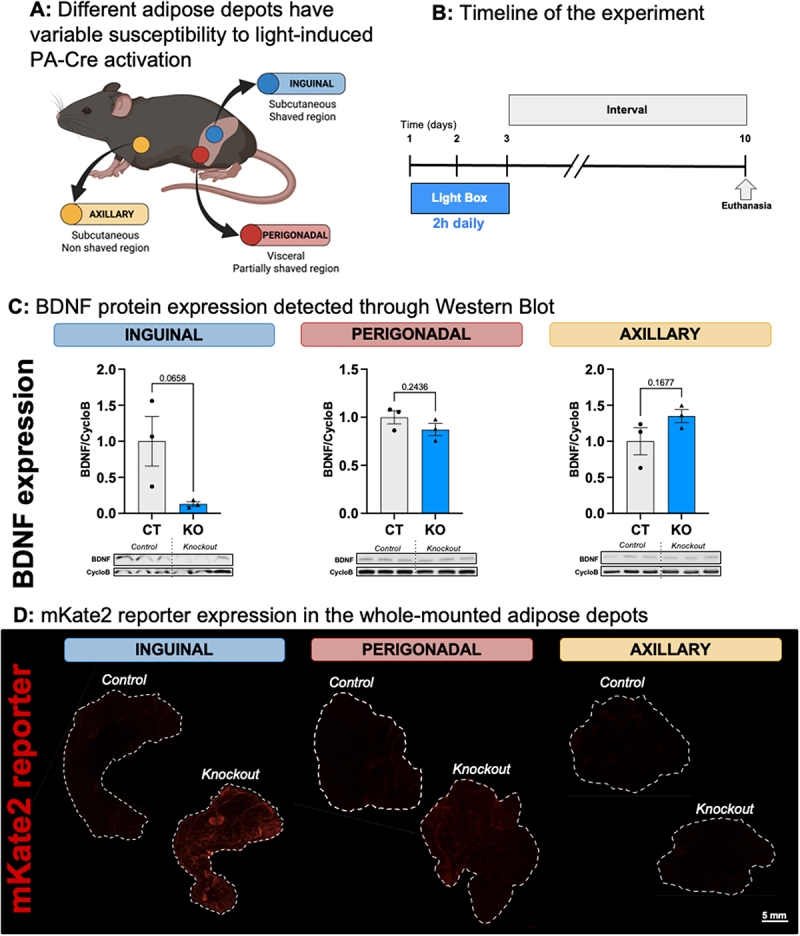
PA-Cre can be induced by a transdermal illumination and black animal fur can be used as a shield to anatomically delimit its activity. A: Different adipose depots have variable susceptibility to light-induced PA-Cre activation depending on their depth and fur coverage. Inguinal adipose tissue is subcutaneous and the skin above it was shaved before light exposure, in BL6 mice with black fur. Perigonadal adipose tissue, a visceral depot, is anatomically deeper compared to the inguinal and was partially below a shaved skin area. The axillary adipose tissue is subcutaneous, but the skin above it was not shaved. B: Timeline of the experiment: the mice were exposed to the blue light in three consecutive daily 2-h sessions and euthanized 7 days after the last session. C: BDNF protein expression assessed through Western Blot in the three adipose tissues dissected. Data is presented as average ± SEM. Grey bars represent BDNFfl/fl mice and blue bars represent KO mice. The groups were compared through a t test, and the *p*-values are reported. D: mKate2 reporter expression in the adipose tissues dissected, by immunostaining. Scale bar: 5 mm.

We tested a non-surgical LED light box stimulus over 3 days to induce PA-Cre only in the inguinal scWAT for whole tissue ablation of the BDNF gene, without knockout in axillary scWAT or visceral WAT depots (which did not receive direct light stimulus). The inguinal scWAT was our main target since this depot is widely studied in the context of adipose tissue ontogeny, physiology and disease [8,42,43], is a health-promoting adipose depot that is prone to neuropathy with obesity/ageing, and is highly translatable to studies of scWAT in humans. The perigonadal tissue was assessed to determine how deep anatomically the PA-Cre was induced in this light box paradigm using a lean mouse with black fur. Axillary scWAT was chosen as a non-shaved region of skin that was exposed to light but shielded by the dark fur, as a control for similar tissue depth as inguinal scWAT (Figure 3(A)).

After shaving above the inguinal scWAT area, control (CT; PA-Cre^−/−^::FLPe^+/-^::BDNF^+/+^) and knockout (KO; PA-Cre^+/-^::FLPe^+/+^::BDNF^fl/fl^) littermates were individually caged for 2 h per day in the light box for 3 consecutive days and were euthanized 7 days after the last session for tissue harvest (Figure 3(B)). We found BDNF protein expression decreased by 87% in the KO inguinal scWAT compared to the CT group (*p*-value = 0.0658). No significant changes were observed in the perigonadal or axillary depots (Figure 3(C)), which served as negative controls. The mKate2 reporter expression, which occurs only with PA-Cre activation, was assessed as validation in whole mount adipose tissues, and was highly expressed in the KO inguinal depot only, as anticipated (Figure 3(D)).

## Discussion

Reduction of BDNF expression by photoactivatable Cre-Lox knockout was successfully achieved in the inguinal scWAT depots via external blue light illumination in awake and freely moving mice placed in a light box 2 h daily for 3 days. A limitation of this system is that any region not covered by fur, such as paw skin, the corneas and retina, are susceptible to light induced PA-Cre activation and should be assessed in systems where the floxed gene is expressed in these tissues. For example, putative effects on the hind paw skin and eyes can affect Von Frey test results and behavioural assessments dependent on the animal’s skin sensitivity and vision, respectively. However, due to the elimination of a surgical intervention to implant a micro-LED, this system ensured a regional subcutaneous adipose tissue gene deletion with minimized stress, improving animal welfare and survival for debilitated mice (e.g. aged, cachexic and obese) or animals in special developmental stages (pregnant and lactating dams, and pre-weaning pups), therefore enabling mechanistic studies in these models. The black fur was proven as an effective shield to anatomically limit PA-Cre activation in this BL6 mouse model, but this system remains to be tested on mice with light coloured fur (white, brown), which may or may not block blue light as effectively.

General aspects of optogenetics experiments should be considered by any investigator undertaking the PA-Cre system. Firstly, blue light was previously shown to induce mitochondrial DNA damage *in vitro* in retinal epithelial cells after exposure to visible light (390–550 nm at 28,000 W/m^2^) for 3 h, but was rescued after 6 h through the natural cellular antioxidant system [44]. Mitochondrial DNA damage was also observed in cultured fibroblasts after blue light irradiation (400–500 nm). They tested several light intensities, and the mitochondrial DNA damage was noted starting at 50J/cm2. It was associated to decreased mitochondrial oxygen consumption rate, ATP production and maximal respiration [45]. We demonstrated successful light induced Cre recombination using a significantly less potent light source than these prior studies, very likely exposing the mice to minimal risk of mitochondrial damage, but this remains to be assessed in future studies. However, animal age and general health status potentially interfere with the animal’s ability to recover from the oxidative stress after blue light exposure. A second consideration is the fact that adipocytes express encephalopsin (OPN3), a 480 nm light-responsive opsin, playing a role in lipolysis and non-shivering thermogenesis [46]. Therefore, acute metabolic effects should be considered in the experimental design when using blue-light activated Cre, and light-exposed flox control mice should be used to control for blue light effects alone.

We have tested this system in lean mice, but obese models would need to be validated in this system given the larger body size and adipose depot size. Light penetration is a potential limitation of this system. There are other photoactivatable Cre mouse lines engineered to be activated by red and far-red lights falling in the wavelength range of 600 to 800 nm [47,48]. This range of wavelength has minimal light attenuation in mouse skin [35], enabling deeper light penetration, and will be useful to control gene expression in tissues deeper than the subcutaneous adipose tissue or with large obese depots. This system can also be refined by inducing cell-specific FLPe expression through viral vector administration. Under these conditions, PA-Cre expression and recombination would occur only in a specific cell type in the illuminated tissue. The adeno-associated virus (AAV) serotype Rec2 was previously validated as adipocyte-trophic [49], and variants on this approach could be useful to target adipocytes in a specific depot using PA-Cre, avoiding whole-body adipocyte cre-lines like adiponectin. Vectors for the transfer of genetic material in the adipose tissue were recently reviewed elsewhere [50].

Overall, we presented proof-of-concept of a simple design to promote light-inducible gene manipulation in distinct subcutaneous adipose depots. By eliminating the step of a surgical procedure for micro-LED implantation, this system allows studies in fragile mice and opens the possibility to adopt gene editing tools with another surgical procedure

## Data Availability

All raw data can be obtained from the corresponding author upon reasonable request.
